# Design of a Flapping Fins Mechanism for Roll Damping of Yachts at Anchor: Kinematic, Hydrodynamic and Structural Study

**DOI:** 10.3390/biomimetics8020144

**Published:** 2023-04-03

**Authors:** Joel Guerrero, Paolo Silvestri, Andrea Canepa

**Affiliations:** 1 DICCA, Politechnic School, University of Genoa, Via Montallegro 1, 16145 Genova, Italy; 2 DIME, Politechnic School, University of Genoa, Via Opera Pia 15, 16145 Genova, Italy

**Keywords:** roll damping, flapping mechanism, kinematic synthesis, structural analysis, hydrodynamic performance, stabilizing fins, zero-speed fins

## Abstract

The design of a flapping fins stabilization system for yachts at anchor (zero speed conditions) is presented in this study. The solution presented in this manuscript took inspiration from a solution proposed for the design of a biologically inspired flapping UAV. Although the application was different, we used the same principles and methodology to design and study the stabilization mechanism discussed hereafter. The proposed system uses flapping fins to damp the roll oscillations of the vessel, and when the stabilization system is retracted, the surface of each of the fins is flush with the hull, thus offering minimum resistance when the ship is in cruise conditions. The unsteady forces of the flapping fins were computed using computational fluid dynamics, and they were used as input to conduct the structural and durability study of the proposed mechanism. The vessel’s response to roll perturbations was also studied, using a multi-body dynamics approach. From the results obtained, and the design specifications defined, it was found that the response of the stabilization system was acceptable, and that the mechanism could withstand the inertial and hydrodynamic loads.

## 1. Introduction

In naval engineering, there is a lot of interest in designing systems able to reduce and control a ship’s undesired roll motion when the vessel is anchored or at zero-speed conditions. The main goal of these stabilization systems is to improve the comfort and safety of the passengers and crew when the boat is anchored and in choppy waters.

For example, if the excitation frequency due to the interaction with the waves is near the boat’s natural frequency, this can cause resonance problems that may be dangerous to the passengers and crew. Undesired and uncomfortable roll motions, due to the interaction with waves, are of concern when operating small-to-medium-sized boats, which are often used for recreational and leisure activities in tourist areas: thus, the importance of attenuating and controlling undesired roll motions at anchor.

In naval engineering, many options are available for stabilization systems, including active and passive anti-roll tanks, passive and active stabilizing fins, flapping fins, and gyroscopic systems, to name only a few. These systems can also be installed in combination, and can be used in small-to-large ships.

Passive anti-roll tanks have become a good means of increasing the comfort of a ship at anchor. Anti-roll tanks can work well when ships are anchored, but the volume of the anti-roll tanks is usually too large, reducing the space available in the vessel; they can also increase fuel consumption, because of added weight. The work by Stigter [1] demonstrated that U-type anti-roll tanks can be quite effective in the reduction of roll motions. These anti-roll tanks can be active or passive. In the case of active anti-roll tanks, the ability to quickly activate them, and the expected low noise generation, make this approach an attractive solution for ships. Because these tanks rely on the resonant response of their content to the roll motion of the ship, careful tuning of the natural period of the ship, and the response of the damping effect of the tank, are important.

One of the first uses of flapping submerged fins was reported in references [2,3]. In these references, the motion of the fins, and their damping effect, were studied. It was reported that the response of the boat, which was contrary to the fins’ motion, differed according to the different phases of the fins’ motion: there was a strong predominance of inertial loading during the starting and stopping phases of the fins motion; then, during the flapping motion, the pressure and viscous forces made the largest contributions.

Another type of fin is the so-called active fins stabilizers, which are appendices that can be extracted from the hull, and can vary their keying angle with respect to the horizontal direction [4]: this is the most widely used stabilization system; however, they do not work well at low speed or when the vessel is anchored (zero speed). The stabilization provided by these devices relies on the physical principle of the generation of a lift force.

More recently, an anti-roll system, based on the Weis-Fogh mechanism of lift generation [5,6], was proposed in references [7,8,9]. The Weis-Fogh mechanism attempts to explain the instantaneous generation of lift produced by the wings of small insects, using the clap-and-filling mechanism: this method can effectively generate large righting forces, to damp the roll motion at zero speed; however, the complexity of the mechanism does not allow for its widespread use, as stated by the authors in references [7,8,9].

Another popular roll stabilization method is the use of gyroscopic systems to contrast the roll motion of the vessel [10,11,12,13,14,15]. Ship-stabilizing gyroscopes, or anti-rolling gyros, may be one of the oldest methods used to stabilize the roll motion of ships at cruise, low speeds, and anchor conditions. The ship’s gyroscopic stabilizer typically operates by constraining the gyroscope’s roll axis, and allowing it to precess either in the pitch or the yaw axes; allowing it to precess as the ship rolls causes its spinning rotor to generate a counteracting-roll stabilizing moment, comparable to that generated by the waves acting on the ship’s hull. Ship-stabilizing gyroscopes basically provide rotational stability via the production of torque. The ability of stabilizing gyroscopes to effectively damp the ship’s oscillations is dependent on a range of factors, including the device’s size, weight, angular momentum, motor, and response time. Anti-rolling gyros have lost favor to hydrodynamic roll stabilizer fins, because of reduced cost and weight; however, there is a renewed interest in these devices for the low-speed roll stabilization of small and medium-sized vessels. Unlike traditional fins, the gyroscope does not rely on the ship’s forward speed to generate a roll-stabilizing moment and, therefore, can stabilize the ship while at anchor.

The solution presented in this manuscript takes inspiration from the mechanism discussed in references [16,17]. The results presented in these references are related to a completely different application—a flying device; nevertheless, the same principles and methodology were used to design and study the zero-speed stabilization mechanism discussed in this manuscript. A zero-speed stabilization mechanism is a mechanism that offers roll damping at low speeds or when the ship is anchored. When the vessel is at cruise, the mechanism is retracted into the hull; therefore, it does not generate hydrodynamic resistance.

By analogy with what was discussed in references [16,17], the proposed stabilization system uses flapping fins (or wings); in this work, however, the main goal was damping roll oscillations rather than producing thrust: that is, to damp the unwanted roll oscillations, the flapping fins needed to generate a counteracting force. Similarly to what was discussed in references [16,17], a flapping kinematics was synthetized. The unsteady forces of the flapping fins were computed using high-fidelity simulations (computational fluid dynamics), and the outcome was used to conduct a structural and durability study of the proposed mechanism. Then, the dynamic response of the ship to roll perturbations was studied, using a multi-body dynamics approach. From the results obtained, it was found that the response of the stabilization system was acceptable, and that the mechanism could withstand the inertial and hydrodynamic loads.

The rest of this manuscript is organized as follows: in Section 2, we briefly review the vessel model, design specifications, and design assumptions; in Section 3, we discuss how the kinematics of the stabilization system was synthetized; Section 4 outlines the computational fluid dynamics solution strategy, where we discuss the results obtained for the vessel’s natural roll frequency, and the hydrodynamic forces acting on the stabilizing fins; in Section 5, the structural and durability analysis of the stabilizing mechanism is presented; in Section 6, we discuss some aspects related to the motor torque requirements; in Section 7, a multi-body dynamics analysis of the vessel and the stabilization system is presented; finally, in Section 8, conclusions and perspectives are outlined.

## 2. Reference Vessel, Design Specifications, and Design Assumptions

**Figure 1:**
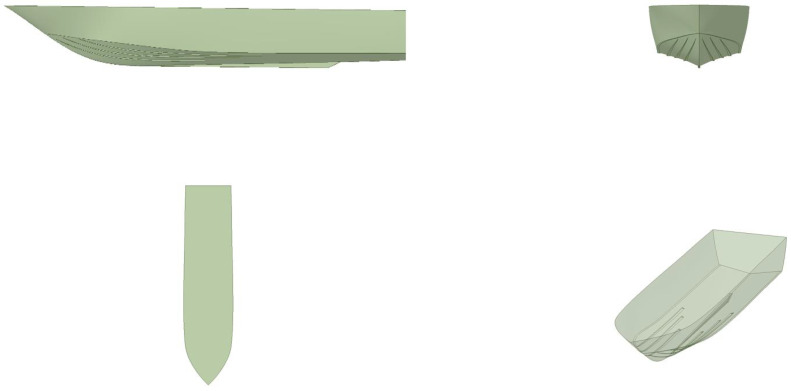
Solid model of the reference hull.

In Figure 1, we depict the solid model of the reference vessel used in this study. We chose to use this hull for two reasons: firstly, it easily fitted within the category of boats used for recreational and tourist activities, where the comfort of the passengers is of utmost importance; secondly, all the physical properties of the ship were known. In addition, the actual vessel was available to conduct physical experiments, take measurements, and fit the proposed mechanism (part of future developments).

In Table 1 and Table 2, we list the main characteristics and physical properties of the vessel, and the main design specifications, respectively. It is important to note that, while the proposed stabilization system is linked to the reference ship, we are confident that the same methodology used in this study can be applied to different vessels with similar dimensions.

As shown in Table 2, the available power ($P_{AVAIL}$) for the stabilization system was constrained to a maximum value of 5.0 kW per fin (10 kW for the two fins used in this study). This design specification was imposed to fulfill power drain, fuel consumption, and noise and vibrations requirements. The design parameter $P_{AVAIL}$ also affected the overall dimensions of the stabilizing fins—that is, the surfaces of the flapping fins that generated the stabilizing force. In this work, the maximum area of a single fin was defined as a design constraint. This value was estimated in function of the design variable $P_{AVAIL}$ and the hull region in which the fins were supposed to be positioned. The actual surface area of the single fin was computed using an iterative method, while the hydrodynamic forces were calculated using computational fluid dynamics (CFD), as described in Section 4; then, these forces were used in the structural analysis, multi-body dynamics study, and motor selection study.

The natural roll frequency of the vessel ($F_{ROLL}$) listed in Table 2 was computed by tilting the boat at different angles, and then measuring the time taken to damp the rolling motion. This study is explained in more detail in Section 4. This frequency was used because it constitutes the main contribution to the roll motion in an untrimmed position and in the absence of external forcing. Furthermore, this frequency is the most significant one in the case of a forced system, as small exciting forces at this given frequency will generate large roll motion responses from the system; therefore, it is generally the most significant frequency content to damp out.

**Figure 2:**
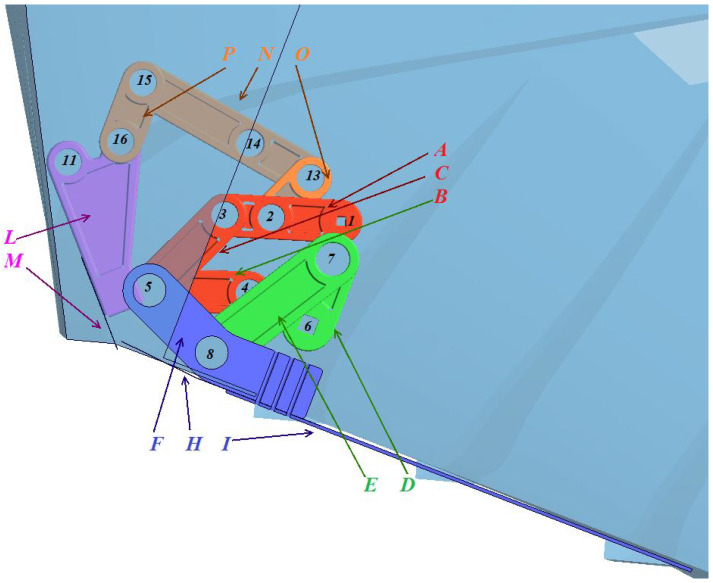
Representation of the extraction/retraction mechanism and the flapping mechanism. In the figure, the elements A, B, C (colored in red); the elements O, P, N (colored in orange); and the elements L, M (colored in pink); represent the structural members of the extraction/retraction mechanism. The elements D, E (colored in green); and the elements F, H, I (colored in blue); represent the structural members of the flapping mechanism.

Hereafter, we list the rest of the design assumptions used during this study:

- The vessel is anchored, and there are no external forces acting on it (i.e., no waves or strong winds).
- The fins are considered to be made of a single part. That is, they are not articulated. Using an articulated fin would have added unnecessary extra complexity to the mechanism and likely with more energy consumption (articulated flapping mechanism is described in references [16,17]).
- The fins are assumed to be rigid.
- The components of the mechanism (crank, rocker, electrical motor, gearbox, levers, and so on), are housed in the hull.
- For the effects of the CFD study, the fins planform is rectangular, their cross-section is uniform and rectangular, and the influence of the fin thickness is negligible.
- When the stabilization system is retracted, the fins surface is flushed with the hull, offering in this way minimum resistance when the ship is in cruise conditions.
- The flapping mechanism and the extraction/retraction mechanism are independent. However, they share a common joint (5 in Figure 2). The extracting and retracting phase of the fins is not discussed in this manuscript.
- The fins motion can be represented by a single degree of freedom, namely, rotation about a given axis. This single degree of freedom is enough to recreate the flapping motion.
- The fins motion is symmetric; that is, both fins move in the same way. However, it is possible to generate an asymmetric motion; therefore, it is possible to make small corrections of the attitude of the boat at zero speed (this is not studied in the current work).
- The proposed solution can be retrofitted in existing hulls, and it should not interfere with other existing stabilization devices.

## 3. Fin Kinematics and Synthesis of the Mechanism

The motion of the flapping fin must be be able to give rise to both hydrodynamic and inertial reactions capable of counteracting the roll motion of the hull. The degrees of freedom needed to achieve this goal are rotational motion around an axis [2,3], and translation motion in the vertical direction [18,19]. For this study, we only considered the rotational motion (which we will call, from now on, the flapping motion), because it is a less complex solution than the translational or combined flapping–translational motions.

The four-bar linkage mechanism is widely used in engineering design [18] . For this study, we use this mechanism, because it provides kinematic pairing, has good behavior at high speed, and does not suffer from wear problems caused by elevated contact forces.

The four-bar linkage kinematic pairs consist of revolute joints only. The absence of prismatic joints assures a reliable and economic structure [18]. As the mechanism must operate in seawater, corrosion is a significant problem; prismatic joints would be affected by precocious wear and by too-frequent maintenance requirements. A revolute joint (as in the four-bar linkage) is less prone to corrosion, and requires fewer maintenance interventions.

The considered mechanism has a single degree-of-freedom (DoF), namely, rotational. The configuration chosen for the four-bar linkage that drives the fin is the crank-rocker type, which has as input the continuous rotational motion of the electrical motor and as output an oscillating moving arm (which induces the flapping motion of the fin). The fin structure is attached to the rockers of the four-bar linkage mechanism. This mechanism was designed according to the Grashov rule [18].

**Figure 3:**
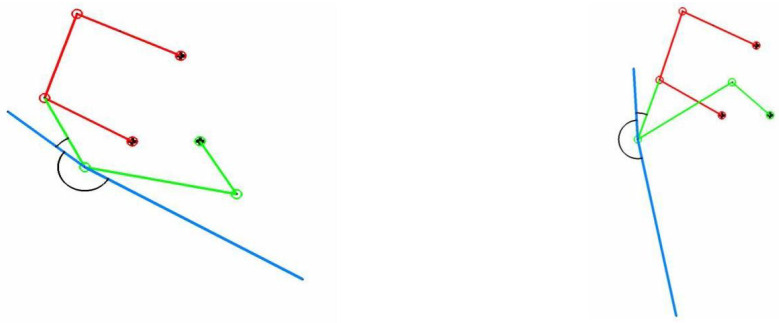
Schematic representation of the extraction/retraction mechanism and the flapping mechanism. (**left** image) mechanism in the bottom-most position (starting phase of the fin motion after the extraction). (**right** image) mechanism in the top-most position. In the images, the red lines represent the extraction/retraction mechanism; the green lines represent the flapping mechanism (the four-bar linkage); the blue line represents the fin, which is attached to the rocker.

In Figure 3, we depict a schematic representation of the mechanism in different positions during the flapping cycle. The extraction/retraction mechanism (red lines in Figure 3) was also designed. However, we do not cover the design process in this work. For more information, the interested reader is referred to reference [20]. To complement Figure 3, in Figure 2 we depict the structural members of the actual mechanism.

In Figure 2, the structural members colored in red (A, B, C), orange (O, P, N), and pink (L, M) represent the extraction/retraction mechanism. The structural members colored in green (D, E) and blue (F, H, I) represent the flapping mechanism, which was the focus of the current work. The four-bar linkage mechanism comprised the following components:

- The crank (D);
- The conrod (E);
- The rocker (F).

The flapping fin is the component H-I and is attached to the rocker (F). The actual roll damping work is done by the fin I. The component H is only relevant when the fin is in the close position. When the fin is closed (retracted position), component H completes the flush closure of the mechanism with the hull. In the figure, the numbers 1 to 16 represent the joining bolts. The design and selection of the joining bolts are not covered in this work. For more information, the interested reader should refer to reference [20].

**Figure 4:**
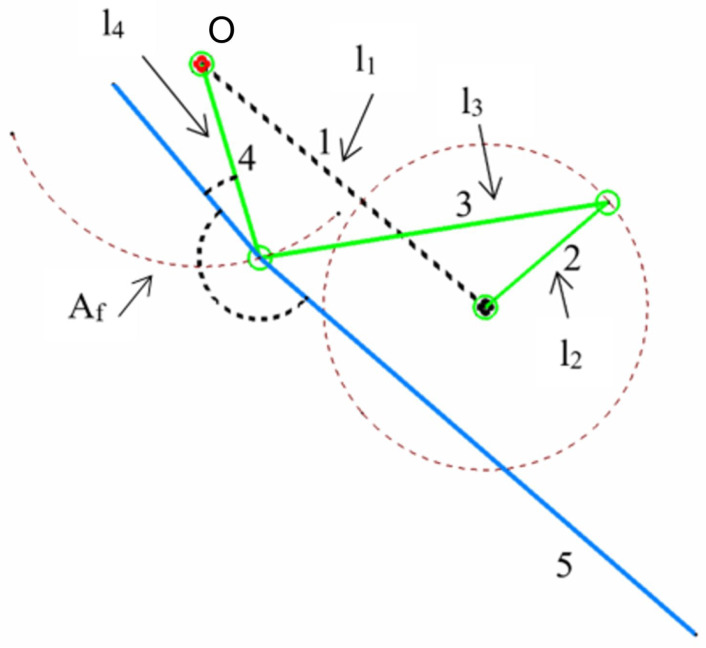
Schematic representation of the flapping mechanism. The arrows indicate the design variables listed in Table 3.

The next step in the design process was to perform the dimensional synthesis of the proposed mechanism: the four-bar linkage. In Table 3, we list the main geometrical variables of the final mechanism. In Figure 4, we depict the schematic representation of these geometrical variables.

The angle $A_{f}$ (refer to Table 3 and Figure 4), is the maximum roll amplitude of the flapping fin. This value was computed using an iterative procedure (as explained later), where we used, as the initial value, $110^{\circ }$. The initial value was obtained by taking into account geometrical constraints and hydrodynamic constraints. To define the geometrical constraints, we took into consideration the following requirements:

- During the flapping motion, the fin should not get too close to the hull, nor hit the hull.
- The fin should not interfere with the extraction mechanism.
- The fin should always remain under the water.
- The flapping angle of the fin should be more than $90^{\circ }$.
- The fin should not get too close to obstacles, such as another boat or a wall.

**Figure 5:**
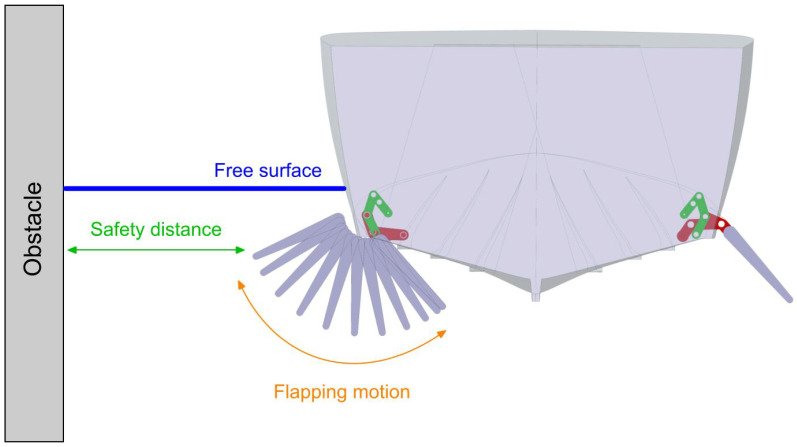
Illustration of the basic layout of the mechanism inside the hull, and some geometrical requirements. The figure is not to scale.

The layout of these geometrical requirements is depicted in Figure 5. Regarding the constraints related to the hydrodynamic behavior, it was essential that the fin should not generate very large vortices that interacted with the hull; in addition, the fin should not get too close to the hull, because it might create additional forces that could destabilize the boat. By respecting the geometrical constraints, it was likely that the hydrodynamic constraints would also be respected.

Using Figure 4 as a reference, the length of the first member of the four-bar linkage is indicated by $l_{1}$. This first member will be henceforth denoted with the subscript 1, and similarly the other parts of the mechanism. In the same figure, the length of the input crank is indicated by $l_{2}$, the length of the conrod is denoted by $l_{3}$, and the length of the rocker is denoted by $l_{4}$.

For the preliminary sizing of the mechanism, we assumed that the angle $A_{f}$ had a strong dependence on the length $l_{2}$ (crank length) and the length $l_{4}$ (rocker), such that:

(1) $$A_{f}=f\left(l_{2},l_{4}\right).$$

**Figure 6:**
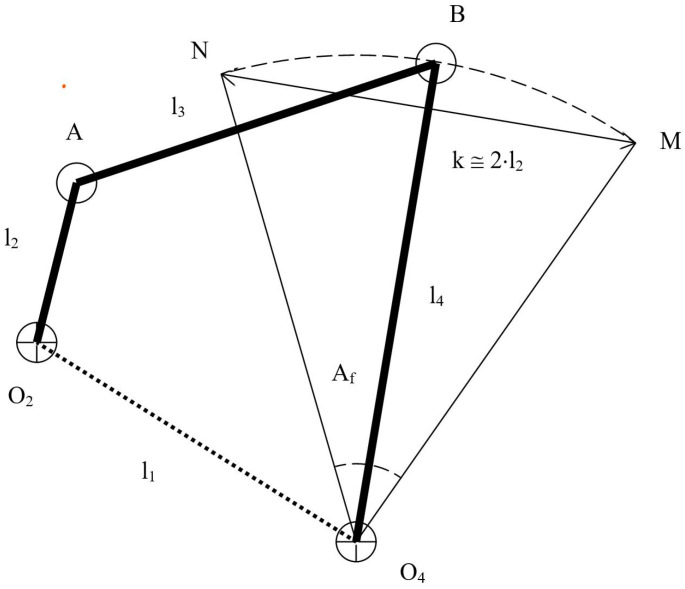
Triangle arising from the geometrical simplification of the four-bar linkage mechanism. In the figure, the base of the triangle ${\mathrm{NMO}}_{4}$ is equal to twice the length of the crank.

In Equation (1), $A_{f}$ is a fixed angle (initial guess, and is equal to $110^{\circ }$), while $l_{2}$ and $l_{4}$ can, in theory, take infinite values. To get an initial approximation of these two lengths, let us use as a reference Figure 6. In the four-bar linkage depicted in this figure, the arc N–M is the stroke of the rocker. This arc can be approximated as a linear length (*k* in Figure 6), as follows:

(2) $$k=2\times l_{2}$$

At this point, and solely based on geometrical arguments, we could find a relation for the angle $A_{f}$ in function of the lengths $l_{2}$ and $l_{4}$, such that:

(3) $$A_{f}=2\times \mathrm{atan}\left(\frac{l_{2}}{l_{4}}\right).$$

By using the initial approximation obtained by Equations (1)–(3), and the geometrical and hydrodynamic constraints previously stated, we can fix the lengths $l_{1}$ and $l_{3}$. Then, by using an iterative procedure, we can find the length of $l_{2}$ and $l_{4}$, which better approximates $A_{f}$.

**Figure 7:**
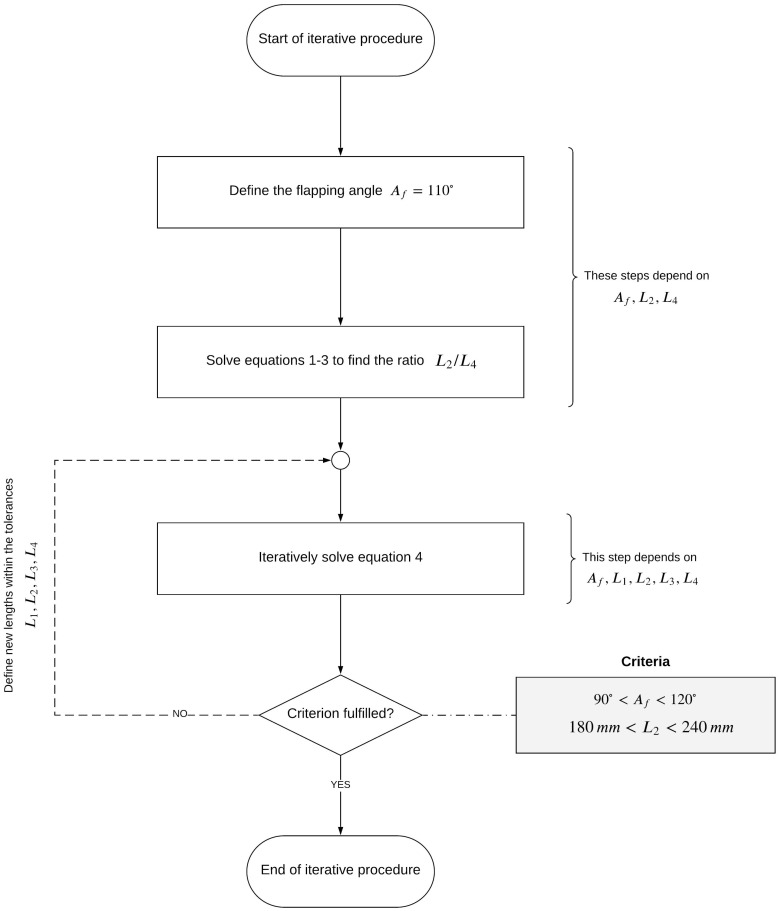
Workflow of the iterative procedure used to approximate the lengths of the elements of the four-bar mechanism.

Let us explain the iterative procedure, using the workflow illustrated in Figure 7. As an initial guess, we used an angle $A_{f}$ equal to $110^{\circ }$. Then, by using Equations (1)–(3) we found the ratio $l_{2}/l_{4}$ (note that Equations (1)–(3) only depended on $A_{f}$, $l_{2}$, $l_{4}$). At this point, and by using Equation (4) and the ratio $l_{2}/l_{4}$, it was possible to find the best combination of design variables ($l_{1}$, $l_{2}$, $l_{3}$, $l_{4}$, $A_{f}$) that satisfies:

(4) $$\begin{array}{c} A_{f}=A_{f}^{Topmost}-A_{f}^{Bottommost}=\frac{360^{\circ }}{2\pi }\times \\ \left(\arccos\left[\frac{\left(l_{4}^{2}-\left(l_{3}+l_{2}\right)+l_{1}^{2}\right)}{2\times l_{1}\times l_{4}}\right]-\arccos\left[\frac{\left(l_{4}^{2}-\left(l_{3}-l_{2}\right)+l_{1}^{2}\right)}{2\times l_{1}\times l_{4}}\right]\right). \end{array}$$

In Equation (4), the values used for $l_{1}$ and $l_{3}$ came from geometrical constraints, and their tolerances were much smaller than those for $l_{2}$ and $l_{4}$; therefore, there was less guessing involved. Regarding the length $l_{2}$, its values could vary between 180 mm and 240 mm. Using these lengths, it was possible to iterate using Equation (4), to find the value of $l_{4}$ that best approximated the angle $A_{f}$. It is important to stress that we were looking for approximated values, so we were not necessarily going to satisfy the requirement of $A_{f}=110^{\circ }$. In Table 3, the final values of this iterative procedure are listed. Note that for the combination of lengths $l_{1}-l_{4}$, the corrected $A_{f}$ was equal to $105.4^{\circ }$.

**Figure 8:**
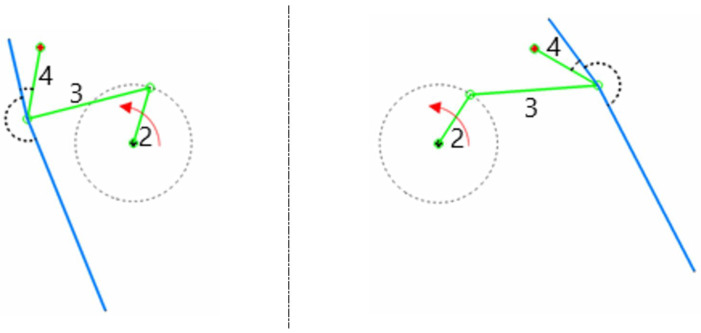
Schematic representation of the left and right mechanisms. The red arrow indicates the sense of rotation of the crank. In the figure, the number 2 represents the crank, the number 3 represents the conrod, and the number 4 represents the rocker. The right mechanism is depicted at the maximum flapping angle, and the left mechanism is depicted at the minimum flapping angle.

So far, a single mechanism has been described. In reality, there are two four-bar mechanisms, positioned symmetrically along the longitudinal axis of the boat, as illustrated in Figure 8. In the same figure, the sense of rotation of the crank is equal for both mechanisms. Therefore, the motion of the fins is in phase. To have a symmetrical motion of the fins, the law governing the angular velocity of the cranks must be in anti-phase. This means that when a fin has reached the maximum flapping angle, the other fin is at the minimum flapping angle. This situation is illustrated in Figure 8, where the right fin has reached the maximum flapping angle, and the left fin has reached the minimum flapping angle.

An electrical motor actuates each mechanism. The motor drives the crank, which rotates at a constant angular velocity, and the fin flaps with a non-uniform angular velocity. This angular velocity evolution is due to the design of the four-bar mechanism (refer to Table 3). Different designs will result in different time evolutions of the angular velocity of the flapping motion of the fin.

**Figure 9:**
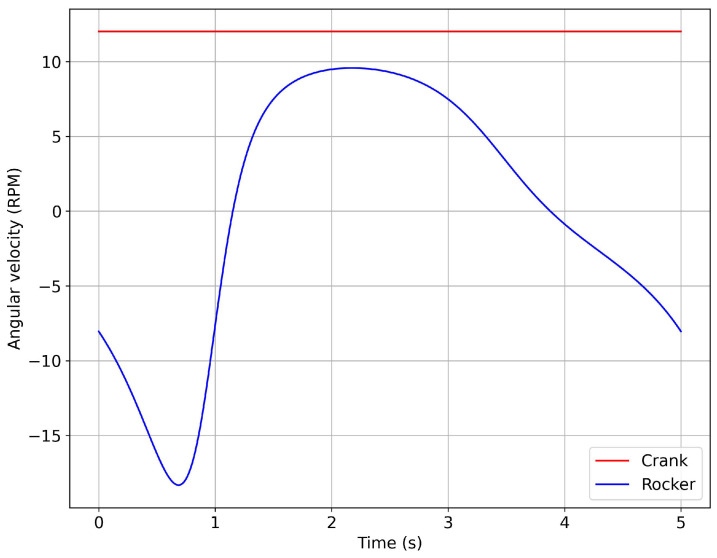
Evolution of the angular velocity during one cycle of the flapping stroke. The blue line represents the rocker–fin component, and the red line represents the crank–motor assembly. The constant angular velocity of the crank–motor gave rise to the variable angular velocity of the rocker–fin.

In Figure 9, we illustrate the scenario mentioned above, where we plot the evolution of the angular velocity for one cycle of the flapping stroke. In this figure, the red line represents the angular velocity of the crank, which is driven by an electrical motor whose output is a constant angular velocity. The blue line represents the evolution of the angular velocity of the fin (which is attached to the rocker) during the up-stroke and down-stroke motions of the flapping cycle. In the next section, we will study the effect that the fin’s angular motion evolution (blue line in Figure 9) has on the hydrodynamic forces.

In this phase of the study, we did not take into account the maximum power constraint of 5.0 kW (refer to Table 2): this constraint will be used in Section 5 and Section 6, to dimension the fin and select motor, respectively.

## 4. Computational Fluid Dynamics Simulations

This section discusses the Computational Fluid Dynamics (CFD) solution strategy, and the outcome of the numerical simulations. An extensive campaign of CFD simulations was conducted, to obtain the model’s natural roll frequency and the hydrodynamic forces acting on the flapping fins. These results were then used in the successive steps: structural analysis (Section 5); motor selection and torque profile (Section 6); and multi-body dynamics study (Section 7).

### 4.1. CFD Solution Method

All the CFD simulations described in this section were conducted using the open-source OpenFOAM library [21,22] (version 8.0). This toolbox is based on the cell-centered finite volume method, and consists of a series of numerical discretization schemes, linear systems solvers, velocity-pressure coupling methods, and physical models that can be used to solve multi-physics problems. To deal with the physics of separated multi-phase flows, we used the volume of fluid (VoF) phase-fraction method to resolve the interface between phases.

In this study, the incompressible, unsteady Reynolds-Averaged Navier–Stokes (URANS) equations for single-phase and separated multi-phase flows were solved using a robust, stable, and high-resolution numerical scheme. The cell-centered values of the variables were interpolated at the face locations, using a second-order centered differences scheme with non-orthogonal corrections for the diffusive terms. The convective terms were discretized using a second-order linear-upwind scheme, and to prevent spurious oscillations, a multi-dimensional slope limiter was used. To resolve the interface between the two phases, a second-order accurate and bounded total variation diminishing (TVD) scheme was used: specifically, we used the van Leer TVD scheme. The least squares cell-based reconstruction method was employed for computing the gradients at the cell centers. The pressure-velocity coupling was achieved through the iterative pressure-based PISO method [23,24]. For turbulence modeling, we used the $y^{+}$ insensitive k−ω SST turbulence model, as described in reference [25]. All the simulations were conducted in unsteady mode, where we used the implicit Crank–Nicolson method to integrate in time the governing equations. The proposed solution strategy resulted in a numerical method that was second-order accurate in space and time.

To handle the moving bodies, a dynamic meshing model was employed [26], where we used mesh diffusion smoothing to deform the mesh. To avoid degenerated cells, manual remeshing was used when the mesh quality was deemed unacceptable. To drive the manual remeshing stage, we monitored two mesh quality metrics thresholds: maximum non-orthogonality and minimum cell volume. The allowable maximum non-orthogonality was set to 85 degrees, and the minimum cell volume was set to 20% of the initial volume of the cell. Then, when any of these metrics were reached, the simulation was stopped, the position of the body was extracted, and a new mesh was generated, using the new position of the body. After the new mesh had been generated, the previous solution was interpolated into the new mesh, and the solution was restarted.

In this study, the forces *F* (*x*, *y*, *z* components) and moments *M* (calculated about a reference position, e.g., the center of gravity) were computed by directly integrating the pressure and wall-shear stress into the surface of the body. As we were dealing with unsteady fluid dynamics, the forces and moments were averaged in time as follows:

(5) $$\bar{F}=\frac{1}{T}\int_{t}^{t+T}F\left(t\right)dt\;\;\;;\;\;\;\bar{M}=\frac{1}{T}\int_{t}^{t+T}M\left(t\right)dt,$$

where T was a reference period, e.g., the period of the roll motion of the hull or the period of the flapping motion of the fins.

### 4.2. Hull Roll Response

**Figure 10:**
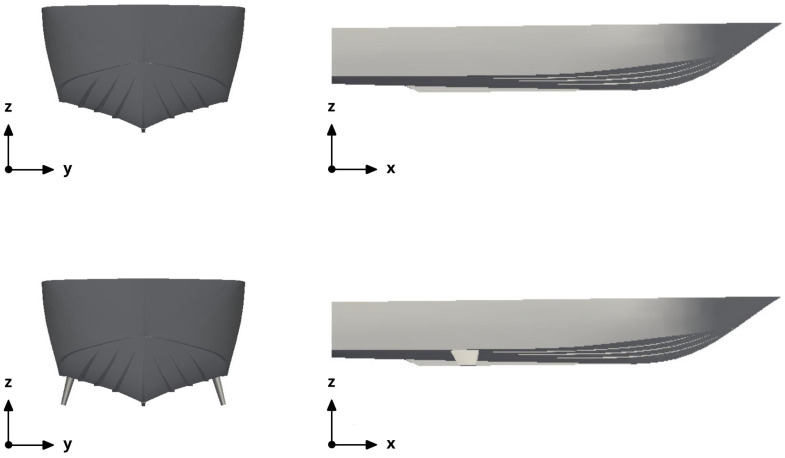
Configurations studied. Top row: hull configuration with no fins. Bottom row: hull configuration with fixed fins.

In this stage, we conducted a campaign of CFD simulations to characterize the roll response of the hull. The governing equations are the incompressible URANS equations for separated multi-phase flows, and we used the volume of fluid (VoF) phase-fraction method to resolve the interface between phases. In Table 4, we list the physical properties of the two phases used in this study, namely, water and air. Two configurations were studied: a configuration with no fins and a configuration with fixed stabilizing fins, as illustrated in Figure 10. The idea of exploring the configuration with fins was to determine if the fixed fins, with dimensions similar to those of the active fins, could dampen the roll motion.

As the scope of this study was to characterize the natural roll frequency of the model vessel, all the simulations were conducted in calm waters (no perturbations) and at zero speed. As we were only interested in the vertical motion (translation along the *z*-axis) and roll motion (rotation around the *x*-axis), rigid body simulations with two degrees of freedom (2DoF) were conducted.

The dimensions of the computational domain used in this study corresponded to five times the waterline length $L_{WL}$ in the x and y directions (approximately 100 m), and two times $L_{WL}$ in the z direction. The main reason for using such a large domain was to avoid or minimize waves reflection at the outer boundaries. In addition, an explicit wave damping source term was used to prevent reflections from the far field boundaries. The mesh used was hexa-dominant, and was made up of approximately 6.5 million elements.

Regarding the dynamic meshing technique, up to a roll angle of 25 degrees, the simulations did not require the use of manual remeshing. During this study, it was found that the flapping fins were too close to the free surface for roll angle values of approximately 20 degrees; thereafter, to fulfill the design requirement that the fins should always remain under the water, we limited the maximum initial perturbation to a value of 15 degrees.

**Figure 11:**
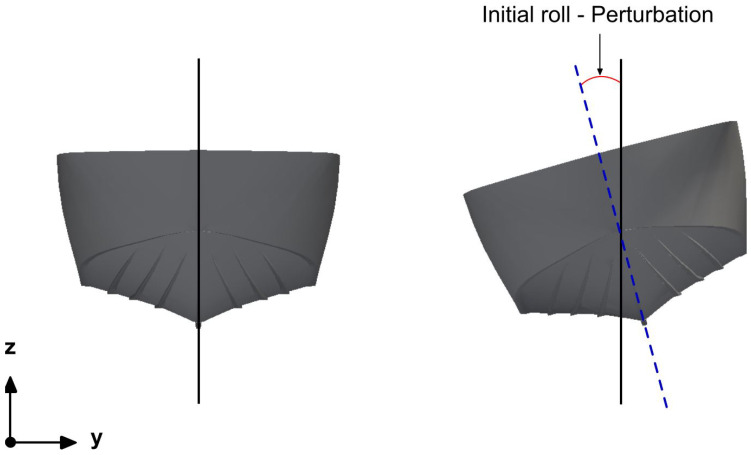
Initial roll angle. The roll angle illustrated in the figure corresponds to 15 degrees.

**Figure 12:**
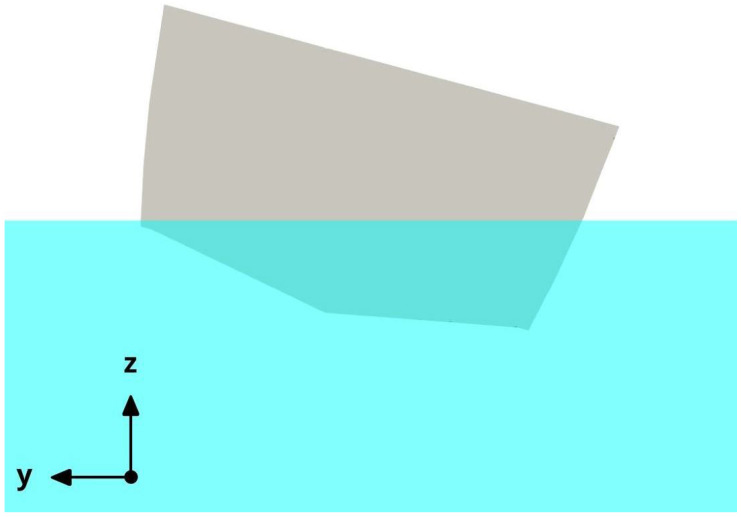
Water level initialization (in cyan) at a roll angle equal to 15 degrees.

To characterize the roll response of the vessel, the hull was set in an initial roll angle (as illustrated in Figure 11), and then the roll angle evolution was measured by using a rigid body motion VoF solver. In Figure 12, we depict the water level initialization and the boat at an initial roll angle of 15 degrees.

**Figure 13:**
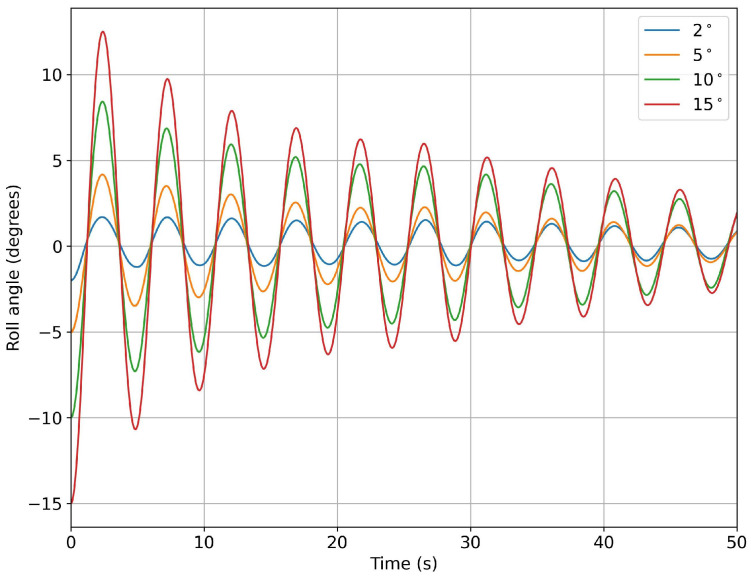
Time evolution of the roll angle at different roll initial conditions for the configuration with no fins.

**Figure 14:**
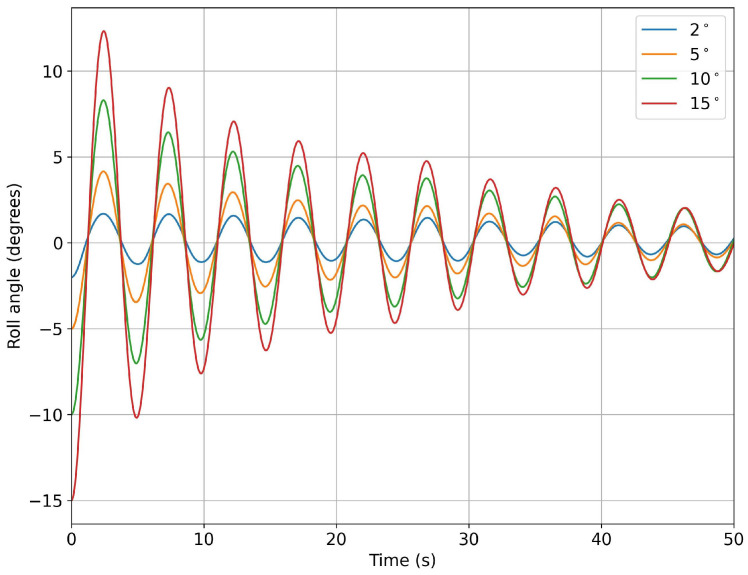
Time evolution of the roll angle at different roll initial conditions for the model with fixed fins.

In Figure 13, we depict the roll angle evolution for the hull configuration with no fins. As can be seen from this figure, the hull had a very clear damping frequency of approximately 0.2 Hz. A similar study was conducted for the hull configuration with fixed fins, where similar behaviors were found, as shown in Figure 14.

**Figure 15:**
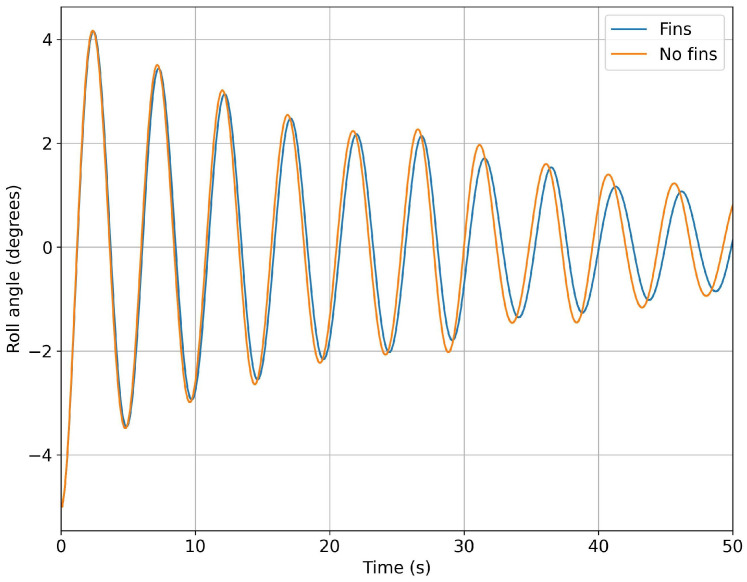
Comparison of the time evolution of the roll angle for the configurations with no fins and with fixed fins: in both cases, the initial roll angle was equal to 5 degrees.

**Figure 16:**
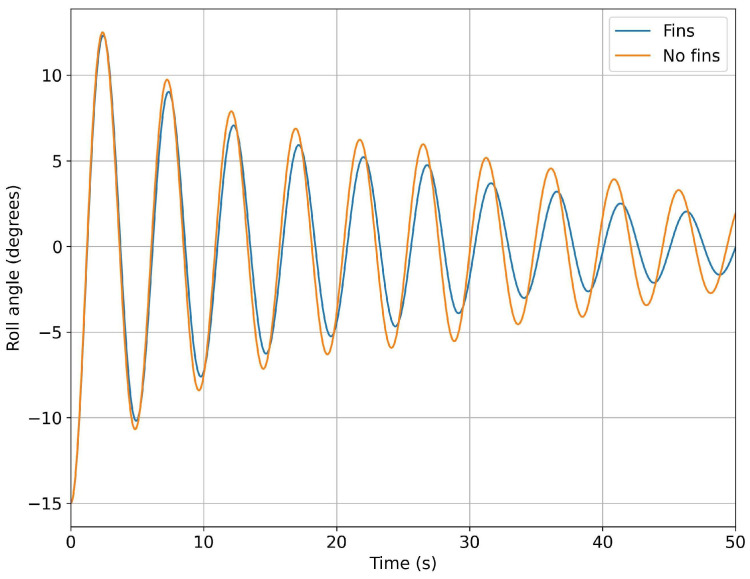
Comparison of the time evolution of the roll angle for the configurations with no fins and with fixed fins: in both cases, the initial roll angle was equal to 15 degrees.

**Figure 17:**
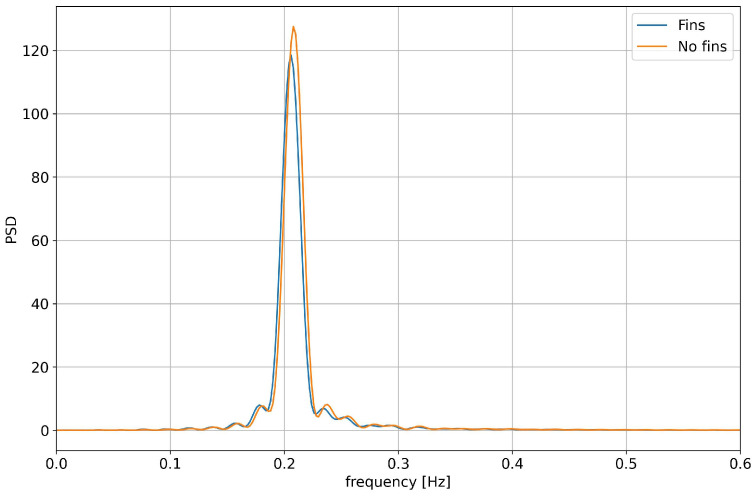
Comparison of the damping frequency for the configurations with no fins and with fixed fins: in both cases, the initial roll angle was equal to 5 degrees.

**Figure 18:**
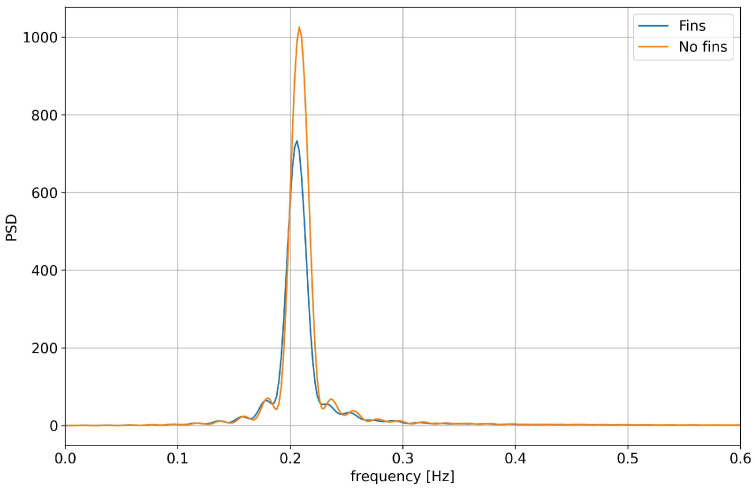
Comparison of the damping frequency for the configurations with no fins and with fixed fins: in both cases, the initial roll angle was equal to 15 degrees.

In Figure 15 and Figure 16, we present a comparison of the roll angle evolution for both configurations (refer to Figure 10) at different initial roll angles. As seen from these figures, the fins helped to dampen the oscillations faster. This damping effect was clearer for high initial roll angles (Figure 16). In Figure 17 and Figure 18, we depict the dominant frequency for both configurations at different initial roll angles. The dominant frequency was computed using a fast fourier transform (FFT) of the time signal of the roll angle evolution at a constant time step. As can be seen from these figures, and disregarding the use of fins, the vessel had approximately the same natural damping frequency.

From this study, it is clear that the use of fixed stabilizing fins has a positive effect on the damping of the roll motion. The natural roll frequency of the hull remains almost the same (approximately 0.2 Hz). This frequency constitutes the main contribution to the roll motion in an untrimmed position and in the absence of external forcing. Therefore, it is the most significant frequency content to damp out.

During this study, we conducted a total of 28 simulations, where we set the vessel at different initial roll angles. In all simulations, the time step was chosen in such a way that the maximum CFL number was never larger than four. Each simulation lasted approximately 36 to 48 h, using 20 cores. All the simulations were run for at least 50 s of physical time.

### 4.3. Forces Acting on the Fins

At this point, we conducted a campaign of CFD simulations, in order to determine the forces acting on the fins at different flapping frequencies. The governing equations are the incompressible URANS equations for single-phase flows, and the working fluid was water (refer to Table 4 for the physical properties).

**Figure 19:**
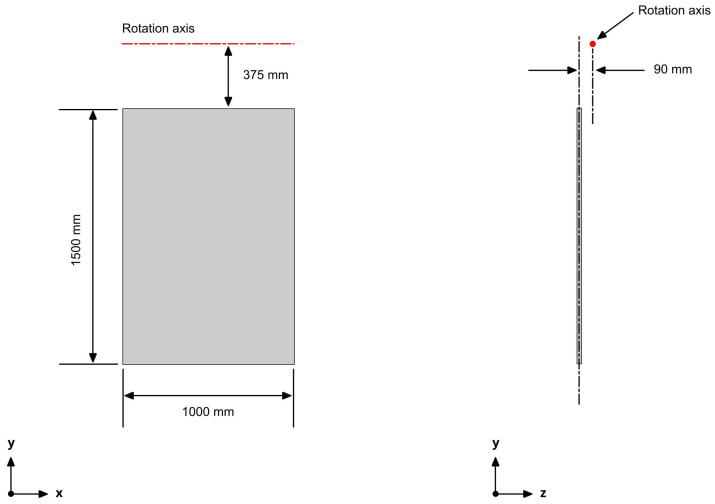
Flapping fin geometry and location of rotation axis. The thickness of the flat plate was equal to 10 mm. The rotation axis corresponded to point 5 in Figure 2. The figure is not to scale.

The flapping fins were modeled as a rectangular flat plate, with the dimensions shown in Figure 19. These dimensions were given as a design condition, and were close to the dimensions of the fixed fins used in the previous subsection. The location of the flapping fins was the same as the location of the fixed fins, which was also a design requirement. The rotation axis depicted in Figure 19 corresponded to point 5 in Figure 2, or point O in the schematic depicted in Figure 4.

The flapping fins oscillate according to the kinematics synthetized in Section 3. To conduct the numerical simulations, the discrete angle and angular velocity evolution were expressed as a trigonometric Fourier series, as follows:

(6) $$y=a_{0}+\sum_{n=1}^{N}a_{n}cos\left(n2\pi ft\right)+b_{n}sin\left(n2\pi ft\right).$$

**Figure 20:**
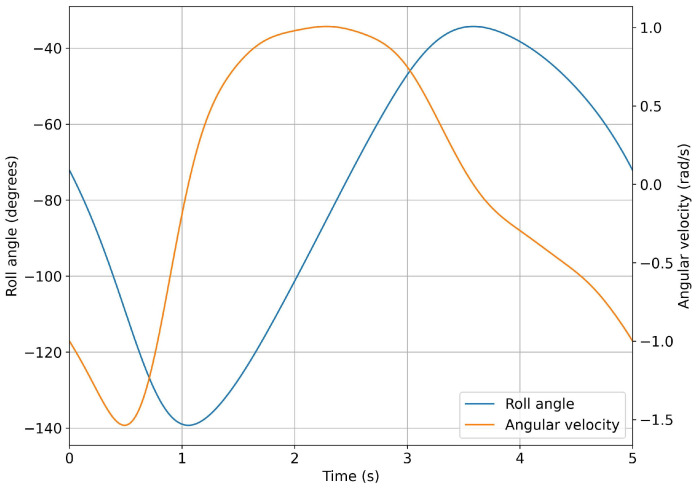
Time evolution of the roll angle and angular velocity for a flapping frequency of 0.2 Hz.

In Equation (6), *f* represents the flapping frequency (in Hertz), and *t*, the time. To define the Fourier series that described the motion, we used eight terms in Equation (6). Table 5 lists the coefficients for a flapping frequency of 0.2 Hz. For the purposes of the numerical simulations, the input value used to define the kinematics was the roll angle; therefore, the coefficients listed in Table 5 were related to the roll angle. Figure 20 depicts the angle and angular velocity evolution for the same flapping frequency.

**Figure 21:**
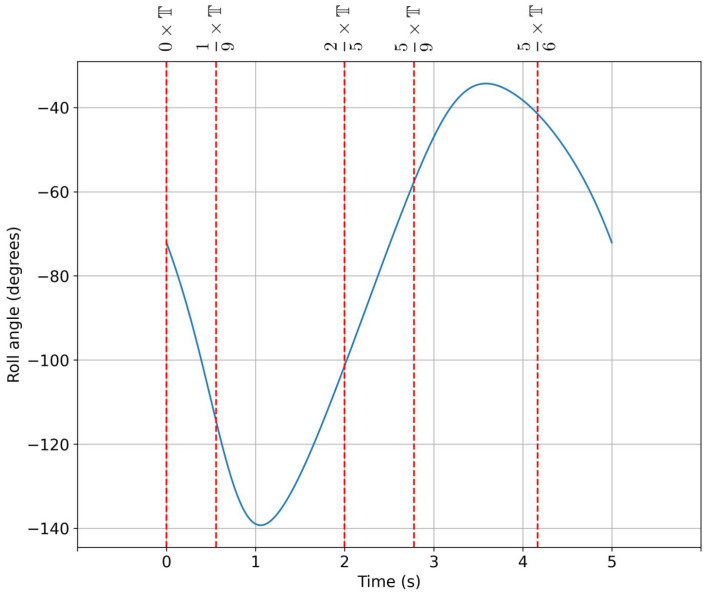
Remeshing periods (vertical lines). The roll angle evolution shown in the figure corresponded to a flapping frequency of 0.2 Hz.

Regarding the dynamic meshing technique, the mesh was subject to large deformations. Therefore, we used manual remeshing. The remeshing frequency was chosen so that the swept roll angle was never more than 40 degrees. In Figure 21, we depict the periods at which the manual remeshing was conducted (the vertical lines). As the motion was prescribed, the five meshes were created a priori, and when the simulation reached the given remeshing period, the simulation was stopped. At this point, the current solution was interpolated to the new mesh, and the simulation was restarted. To avoid user intervention, this task was automated, using shell scripting. The mesh used in this study was made up of approximately 1.5 million tetrahedral elements. This cell type was used as it diffuses better the mesh deformation in the domain.

In Table 6, Table 7 and Table 8 we summarize the mean value, maximum value, and the minimum value for different oscillating frequencies. All the statistics were computed during the third period of oscillation, where the forces showed statistically steady behavior. From these tables, we can see that for oscillating frequencies higher than 0.333 Hz, the forces generated (mean force and minimum and maximum peaks) were too high for the fin dimensions and material selected. These observations were confirmed during the structural analysis of the flapping fins. It is important to mention that the stresses on the crank, rocker, and conrod were the limiting factors of the mechanism: that is, these components were likely to fail long before the fin; therefore, the structural and durability analyses of the fins are not discussed.

**Figure 22:**
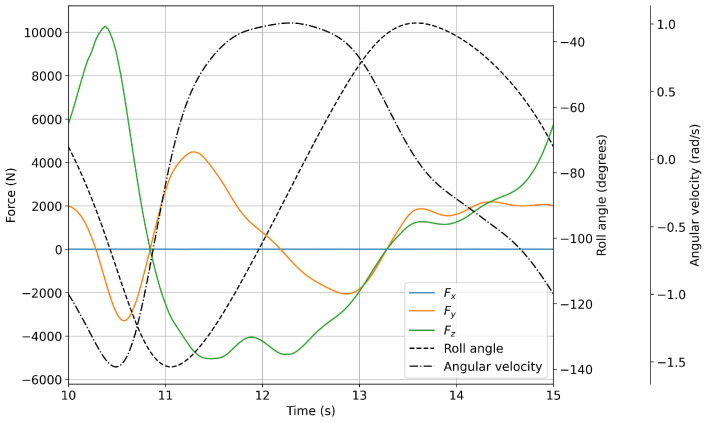
Forces evolution during a flapping period (continuous lines). The dashed and dash–dot lines represent the roll angle and angular velocity, respectively. The flapping frequency was equal to 0.2 Hz.

**Figure 23:**
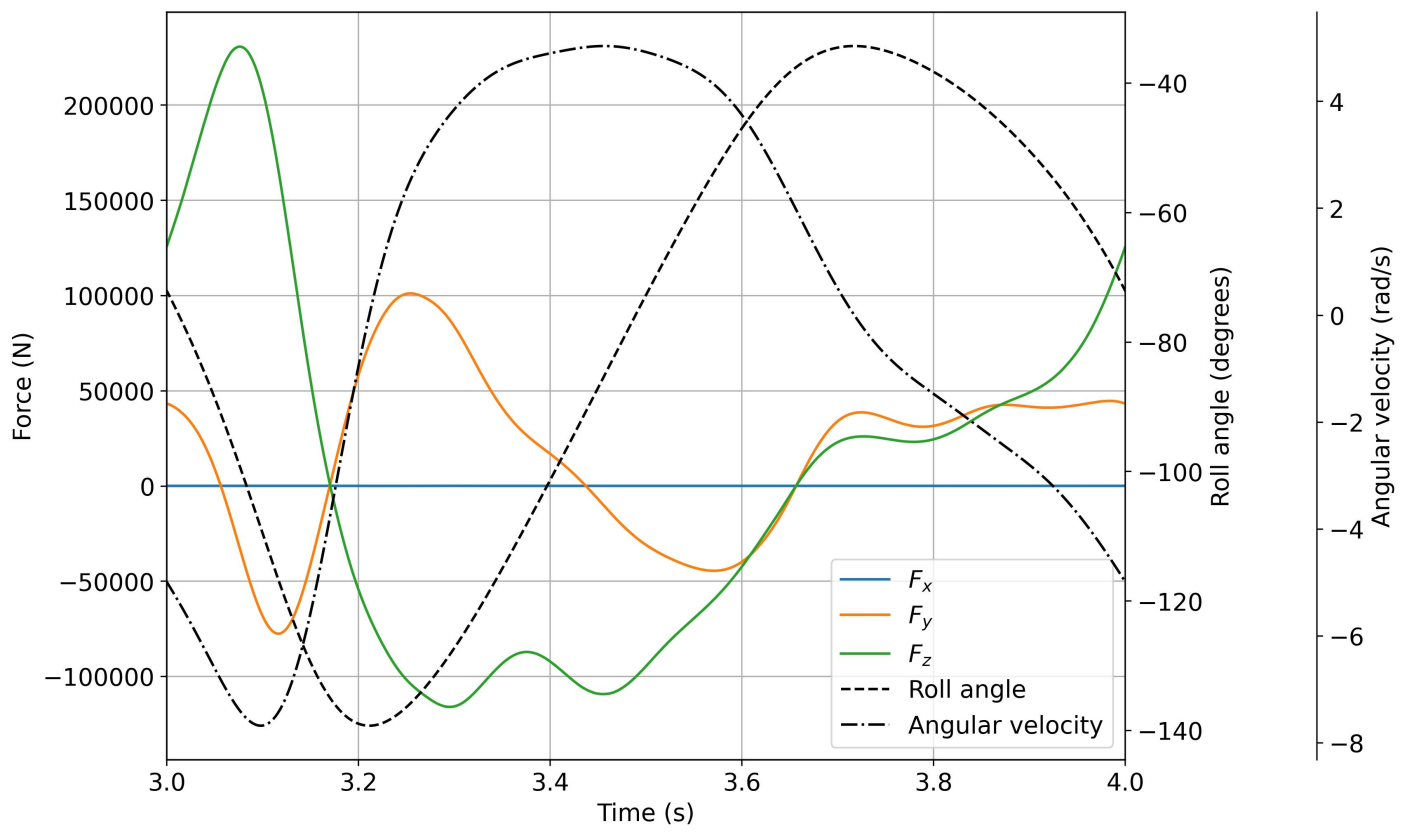
Forces evolution during a flapping period (continuous lines). The dashed and dash–dot lines represent the roll angle and angular velocity, respectively. The flapping frequency was equal to 1.0 Hz.

In Figure 22 and Figure 23, we plot the forces evolution for a flapping frequency of 0.2 Hz and 1.0 Hz, respectively. As can be seen from these figures, all the cases show a similar time evolution for the force components. The difference between the simulations relies on the fact that the forces that act on the fin are smaller for lower oscillating frequencies.

During this study, we conducted a total of 12 simulations. One set of simulations with a maximum CFL number of one, and the other set of simulations with a maximum CFL number of four. The difference in the outcome of both campaigns was less than 5%. All the results discussed in this section correspond to a CFL number of one, and each simulation lasted approximately 56 to 72 h, using 20 cores. All the simulations were run for at least three flapping periods.

Finally, it is important to note that the results presented in Section 4.2 and Section 4.3 were used as input for the structural analysis (Section 5), motor selection and torque profile (Section 6), and the multi-body dynamics simulations (Section 7).

## 5. Structural Analysis

In this phase of the study, we determined the sizing of the structural components, in terms of both maximum stress and fatigue life based on the reliability requirements. The system in question can be divided into the fin body and the associated drive mechanism (crank–conrod–rocker). In actual operation, the two systems interact; however, it is assumed that the structure of the fin is sufficiently rigid, such that it can be considered dynamically separated from the rest of the system. This hypothesis enabled the sizing of the two elements separately, taking into account their interaction through the definition of appropriate static forces applied to the structural joints. From this point on, and unless otherwise specified, when we refer to the mechanism, we deal with the crank–conrod–rocker assembly (drive mechanism).

The stress, deformation, and the durability analysis presented in the next subsections, were conducted using the structural solver LMS Virtual Lab [27,28]. The loads used to perform the structural analysis were obtained at a flapping frequency of 0.2 Hz. This frequency corresponded to the natural roll frequency of the boat, and it was obtained from the CFD study explained in Section 4. In addition to these loads, we also applied inertial loads to the fins, which were obtained from the LMS Virtual Lab. It is worth noting that, for the mechanism to operate at 0.2 Hz, the crank must rotate at an angular velocity of 12 RPM.

### 5.1. Stress and Deformation Analysis

This analysis was carried out assuming a load equal to the peak hydrodynamic force uniformly distributed on the fin. The time-dependent hydrodynamic forces were obtained from the CFD study explained in Section 4. In addition to the frequency of 0.2 Hz. (which corresponded to the natural roll frequency of the vessel used in this study), we also stressed the components at higher flapping frequencies. The idea was to determine what was the maximum flapping frequency that the mechanism could withstand, such that if we could damp higher frequencies, the mechanism could be used in different vessels with different natural roll frequencies or under different operational conditions (waves, strong winds, loading/unloading, and so on). Additionally, we also considered the inertial loads’ contribution during the flapping cycle. The inertial loads were computed assuming a uniform material distribution, where we used standard steel as the material for the mechanism (refer to Table 9 for a description of the physical properties).

**Figure 24:**
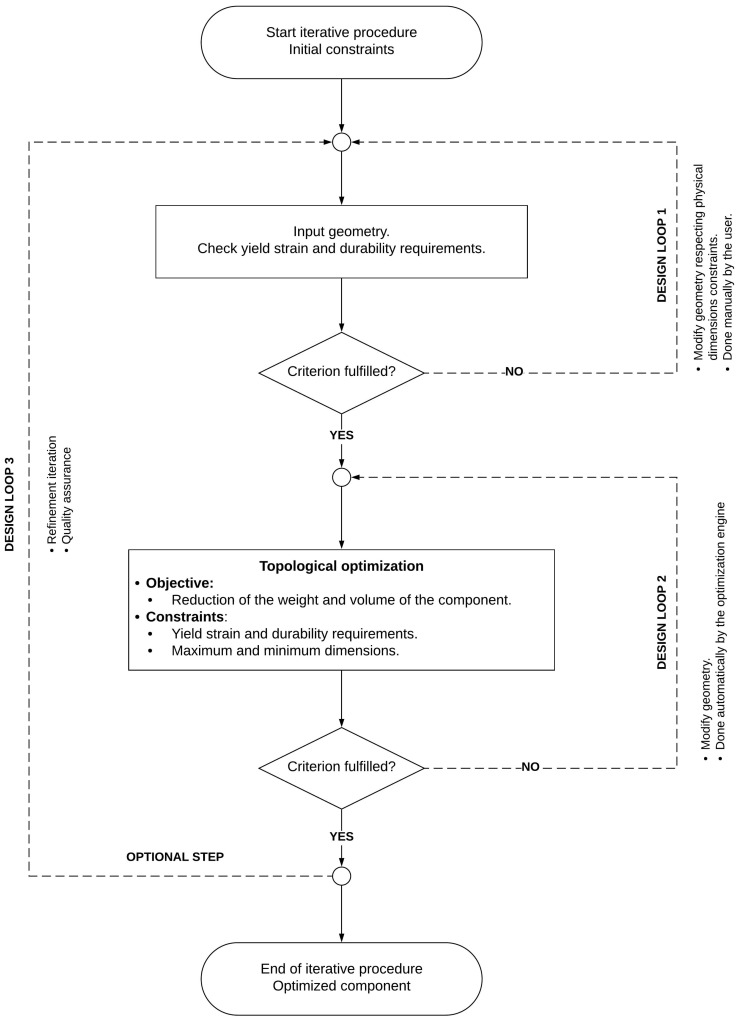
Workflow used for the sizing and structural design of the components of the mechanism.

In Figure 24, we illustrate the design workflow used for the sizing of the components that made up the proposed mechanism. The workflow starts by using the geometrical and design requirements specified in the previous sections. The first design loop is an iterative process done manually by the designer. In this design loop, an initial sizing of the mechanism is proposed, and then the designer manually iterates, to obtain better solutions. It is important to stress that the new solutions must occupy a minimum volume in the hull, be light, and withstand the loads. During each design iteration, the yield strain and durability requirements are monitored. If the new solutions fulfill the design and structural requirements, the designer can move to the second design loop, which involves topological optimization. Again, this is an iterative process, but this time it is done automatically by the structural solver (LMS Virtual Lab [27,28]). The main goal of the topological optimization is to reduce the weight of the structural components and, at the same time, satisfy the yield strain, durability, and physical dimensions requirements. If this criterion is met, the designer can end the design workflow or do another design iteration (third design loop), to get an even better design, or for quality assurance.

In Table 10, the structural analysis results are reported at a frequency of 0.2 Hz. As shown in this table, the maximum stress of each component was lower than the yield stress of the selected material; hence, the structural design was suitable to work under the given operating conditions. For completeness, we also show the maximum displacement and the ratio between the yield strain and the maximum stress on the component.

**Figure 25:**
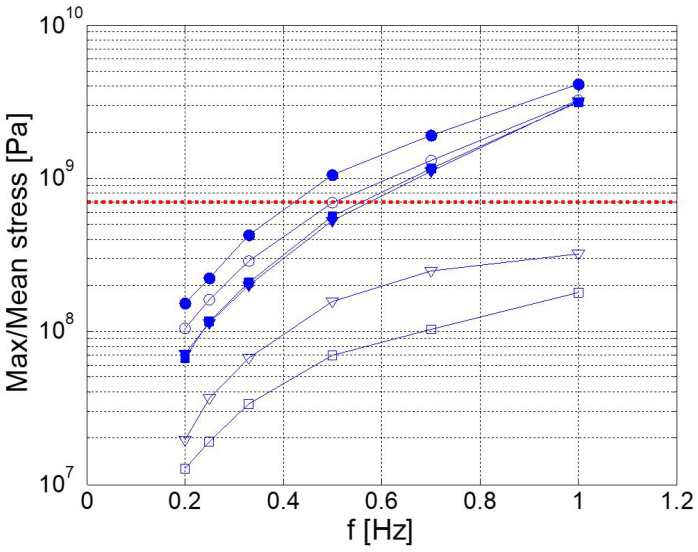
Maximum and mean stresses on each component at different frequencies. In the figure, the circles represent the crank, the triangles represent the rocker, and the squares represent the conrod. The shaded symbols show the maximum stress, and the empty symbols show the mean stress. The dotted red line represents the upper threshold of the yield stress.

In Figure 25, we plot the maximum and mean stresses on each component at different frequencies. The maximum and mean stresses were computed during the whole flapping cycle, using the equivalent von Mises stresses [18,27]. In this figure, the horizontal red line represents the yield stress. Above this line, the components will fail. As can be seen, at the design frequency (0.2 Hz), the stresses on each component were well below the yield stress. Then, as we increased the frequency, up to a value of 0.5 Hz, the mechanism withstood the loads. Above 0.5 Hz, the first component to fail was the crank, followed by the conrod, followed by the rocker.

**Figure 26:**
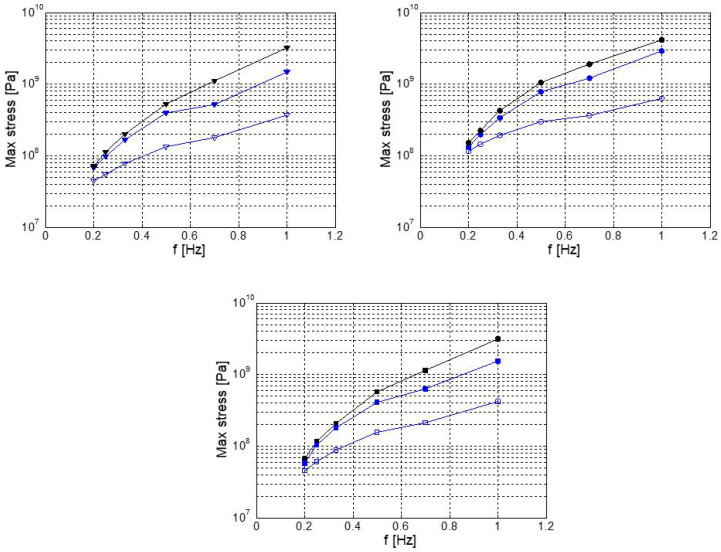
Maximum stresses on each component at different frequencies. The circles represent the crank, the triangles represent the rocker, and the squares represent the conrod.

In Figure 26, we depict the maximum stresses due to the total loads (hydrodynamic plus inertial loads), the individual hydrodynamic loads, and the individual inertial loads on each component of the mechanism at different flapping frequencies. As expected, the hydrodynamic loads were much larger than the inertial loads. Interestingly, the stresses due to the inertial loads were always below the yield stresses.

**Figure 27:**
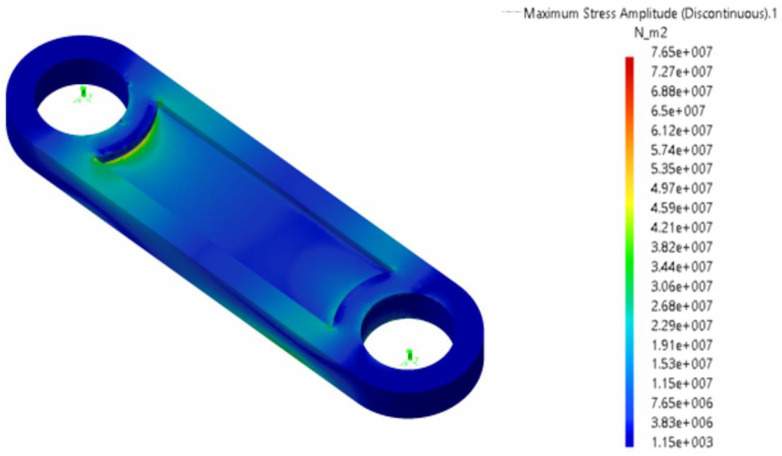
Maximum stresses (Pa) on the conrod during the flapping cycle. Flapping frequency f = 0.2 Hz; maximum stress in the figure is equal to $7.65\times 10^{7}$ Pa.

**Figure 28:**
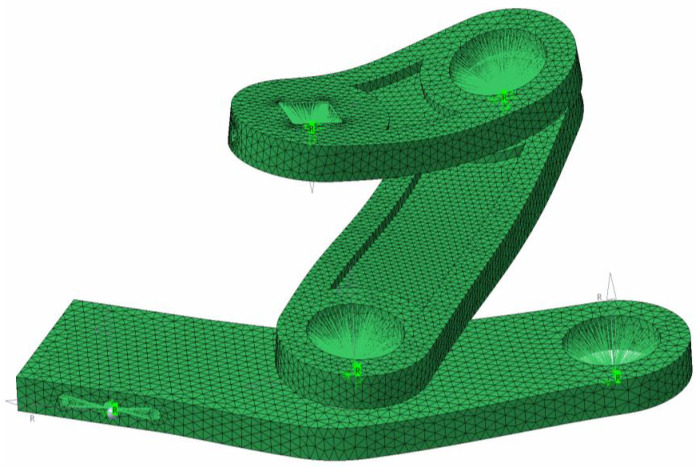
Maximum deformation of the crank–conrod–rocker assembly, amplified by a factor of 100. Flapping frequency 0.2 Hz. The mesh used to conduct the structural analysis is also depicted.

For completeness, in Figure 27 we depict the maximum stresses during the flapping cycle on a single component of the mechanism (the conrod), at a frequency of 0.2 Hz. In this figure, we can evidence the regions that were more stressed and likely to fail. It is important to emphasize that the stresses depicted in this figure represent the maximum local stresses distribution during the flapping cycle. Finally, in Figure 28, we depict the maximum deformation of the crank–conrod–rocker assembly, amplified by a factor of 100.

### 5.2. Durability Analysis

Taking into account the harsh conditions in which the mechanism operates, notably the large hydrodynamic and inertial dynamic loads, as well as the stresses and vibrations to which the components are subjected, it is of interest to proceed with more advanced studies, such as fatigue or durability analysis.

Durability analysis enables the detection of the deterioration of material due to repeated cyclic loading. For most machinery, the time before the first crack appears is much longer than the period from onset to propagation and actual fracture: this means that the useful life of a component is roughly the same as the period of time before cracks appear. The LMS Virtual Lab enables an estimate of crack initiation life, via a mechanics-based analysis of the stresses (or the strains) in the structure, and of the material properties [27,29,30]. Two basic approaches are available in the software: the stress-life approach and the strain-life approach. The stress-life approach, adopted for the present analysis, assumes that all stresses are below the material’s elastic limit at all times: the approach is suitable when applied stresses are nominally within the elastic range, and the number of cycles to failure is large.

To conduct the durability analysis, the software requires fatigue data, in the form of curves of stress against cycles to failure (which we will call N) obtained from specific tests on standardized smooth specimens. To obtain this information, the database available in the LMS Virtual Lab [27] was used. To evaluate the fatigue behavior of the virtual prototype, the loads on the different parts were assessed by means of a multi-body simulation [29]. The model also requires the fatigue behavior parameters for the material, stress/strain curves, and Wohler curves [18]. For the computation of the stresses, we adopted the critical plane, Goodman correction method [18].

We considered different operational conditions of the mechanism, characterized by flapping frequencies ranging from 0.2 Hz to 0.5 Hz, with the hydrodynamic forces evaluated at the center of pressure. Before the fatigue analysis of each part of the mechanism, an analysis was performed to obtain the distribution of the maximum stresses (calculated from the mono-axial equivalent stresses), thus providing a reliable indication of the most critical zones that might be subject to fatigue. In addition, this study yielded results, in terms of durability and fatigue life, these being an indicator of the operating conditions prior to the occurrence of a fatigue crack. This quantity is expressed as multiples of the cycle simulated in the multi-body analysis, corresponding in the case under examination here to the complete flapping motion of the fin.

**Figure 29:**
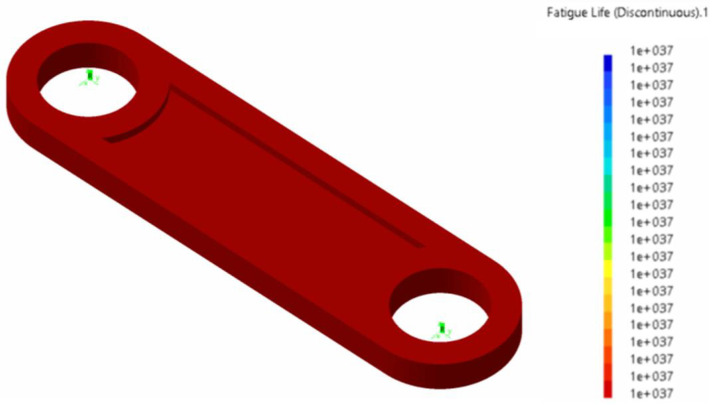
Fatigue life in cycles for the Conrod at a flapping frequency equal to 0.2 Hz. In the figure, the number of cycles is very large, which indicates infinite durability.

In Figure 29, we show the durability results for the conrod, which was considered the most critical part of the mechanism (in terms both of maximum stress and durability). The behavior remained acceptable at the design flapping frequency of 0.2 Hz: that is, the maximum stresses were below the yield strain, and the durability was infinite. At a flapping frequency of 0.5 Hz, the maximum stresses were still below the permissible limit, but the fatigue behavior of the conrod deteriorated drastically, and the maximum number of cycles to fatigue was less than $10^{5}$: from a durability point of view, this was not acceptable.

**Figure 30:**
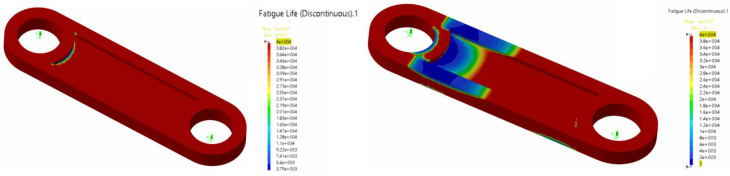
(**left** image) fatigue life in cycles of the conrod at a flapping frequency of 0.25 Hz (infinite life). (**right** image) fatigue life of the conrod at a flapping frequency of 0.5 Hz (finite life). The component is likely to fail in the blue region at less than $10^{5}$ cycles.

In Figure 30, we depict the results at a flapping frequency of 0.25 Hz and 0.5 Hz. Analyzing the fatigue behavior of the conrod at these frequencies, the analysis forecast fatigue life values for the conrod of around $10^{9}$ cycles for a flapping frequency of 0.25 Hz, which was acceptable, but the component was starting to show deterioration due to fatigue (blue regions in Figure 30). Under these conditions, it is strongly recommended to conduct preventive maintenance and periodic non-destructive testing, to check for cracks. On the other hand, for a flapping frequency of 0.5 Hz, the analysis forecast a limited life, well below $10^{5}$. The component would likely fail first in the regions where the life cycle was lower (blue regions). Under these conditions, the life cycle of the conrod was not acceptable, disregarding the fact that the maximum stresses were lower than the yield strain (refer to Figure 25).

**Figure 31:**
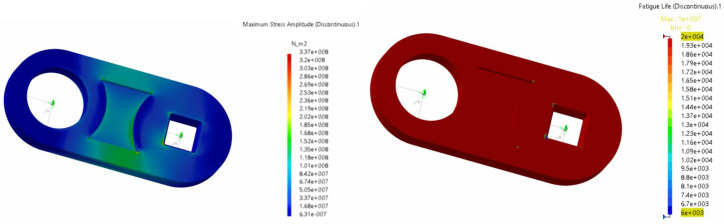
Maximum stresses and fatigue life for the crank at a flapping frequency of 0.25 Hz. (**left** image) maximum stresses (maximum stress $\approx 3.4\times 10^{8}$ Pa). (**right** image) fatigue life in cycles. Note that the critical zones for fatigue life emerge in the area of the notches around the main pin.

In Figure 31, we show the maximum stresses and durability results for the crank at a flapping frequency of 0.25 Hz. As shown in the figure, the maximum stresses were still below the permissible limit, but minimal critical zones for fatigue life emerged in the area of the notches around the main pin.

**Figure 32:**
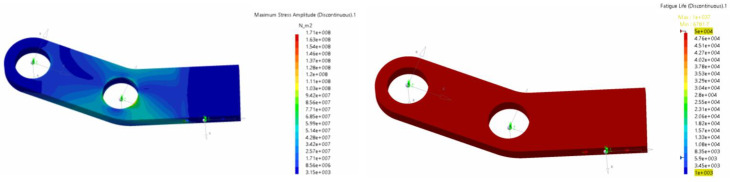
Maximum stresses and fatigue life for the crank at a flapping frequency of 0.25 Hz. (**left** image) maximum stresses (maximum stress $\approx 1.7\times 10^{8}\;\mathrm{Pa}$). (**right** image) fatigue life in cycles. Note that a fatigue limited zone appears in the region where the rocker is connected to the fin.

Figure 32 shows the maximum stresses and fatigue analysis performed on the rocker at the design frequency of 0.25 Hz. At this frequency value, the component still did not present any particular critical areas, with the sole exception of a very limited zone, where the connection to the fin was located. In this same region, the stress analysis revealed a peak.

**Figure 33:**
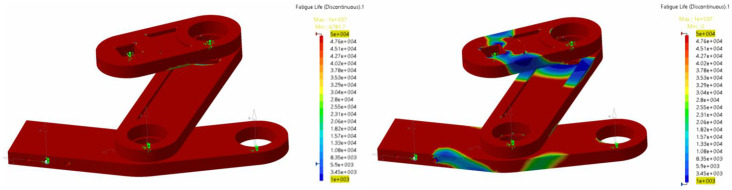
Fatigue life of the crank–conrod–rocker assembly (drive mechanism) for a flapping frequency of 0.25 Hz (**left** image) and a flapping frequency of 0.5 Hz (**right** image).

Figure 33 shows the results of the fatigue life for the drive mechanism (crank–conrod–rocker assembly) at two frequencies: 0.25 Hz and 0.5 Hz. Evaluating the mechanism globally from the fatigue life point of view, the most critical conditions arose in the zones around the pins. Some zones of the rocker, and near the pivot points of the conrod, would appear to give rise to critical issues due to notches and stress concentrations. It is important to note that the right image in Figure 33 corresponded to a high flapping frequency case (0.5 Hz), which represented the most adverse operating conditions of the cases studied during the durability analysis. Operating at lower frequencies significantly reduced the total loads and consequent improvement in fatigue behavior. Finally, from this figure, we can also evidence that the most critical component from the fatigue life point of view was the conrod.

Summarizing the results obtained in this section, we can say that the mechanism can withstand the total loads and has a very high fatigue life when operating at the design frequency of 0.2 Hz. We also found that we could increase the operating frequency up to 0.25 Hz without deteriorating the structural integrity of the mechanism. Operating at frequencies larger than 0.25 Hz will result in shorter fatigue life, even though the maximum stresses are lower than the yield strain.

Finally, as the fins are not subjected to structural criticalities related to maximum stresses and fatigue phenomena, as for the case of the flapping mechanism (crank, conrod, rocker), we do not discuss the structural and durability analysis of the fins.

## 6. Motor Selection and Torque Profile

To determine the torque that the motor had to supply to the mechanism, several simulations on the selected configuration were conducted. Hereafter, we present the results only for the nominal frequency of 0.2 Hz, which corresponded to the natural roll frequency of the vessel. The simulations were conducted using the structural solver LMS Virtual Lab [27], and the methodology was similar to the one used in Section 5.

**Figure 34:**
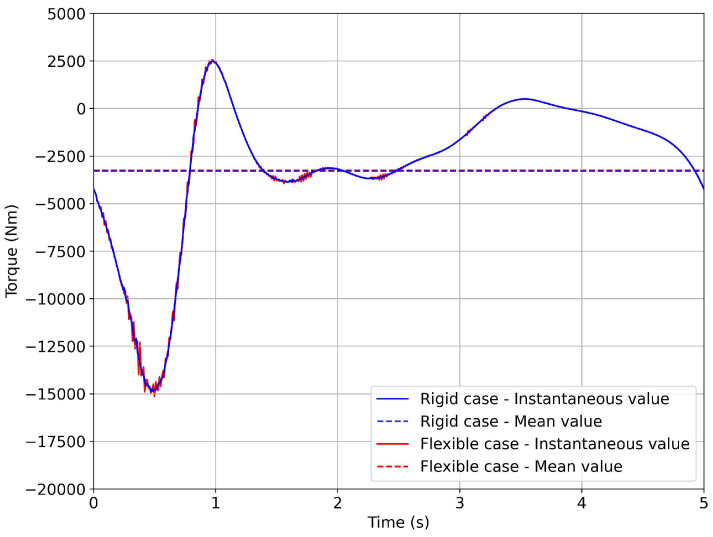
Torque evolution of the motor for one flapping cycle. The blue line represents the rigid case (design condition) and the the red line represents the elastic case. The mean torque in both cases was approximately equal to 3500 N·m (absolute value), and the peak torque was approximately 15,000 N·m (absolute value).

In Figure 34, we show the torque profile evolution. The torque profile for the elastic case is also plotted in this figure. As can be seen, the elasticity of the mechanism introduced a small degree of oscillation in the torque profile, when compared to the rigid case. Elasticity can also introduce hysteresis, which was not seen in this case.

**Figure 35:**
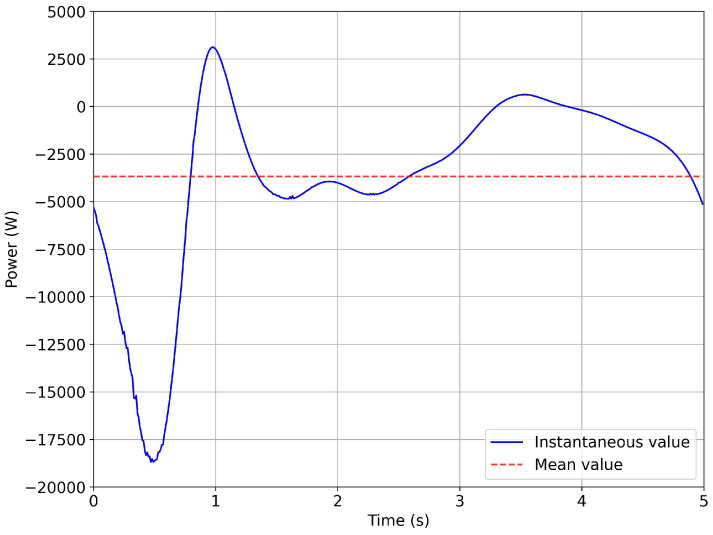
Instantaneous power evolution and mean power of the motor for one flapping cycle. The mean power was equal to 3.8 kW (absolute value). The peak power was approximately 18 kW (absolute value).

In Figure 35, we depict the instantaneous power evolution, which was computed as follows:

(7) Power=Torque×ω.

As can be seen in Figure 35, the average power was less than 5.0 kW, which satisfied the design requirements (refer to Table 2). At this point, an electrical motor fulfilling the design and operational requirements could be selected from a commercial catalog. It is worth noting that the peak value of the torque of the motor was approximately 15,000 N·m (absolute value), with a consequent instantaneous peak power value of around 18kW (absolute value), as depicted in Figure 35. This instantaneous power peak could be reduced to lower values by adding a flywheel.

## 7. Dynamic Analysis of the Vessel and Stabilization System Using a Multi-Body Dynamics Approach

This section complements the mechanism previously designed. Here, we studied the effectiveness of the stabilization system in damping the rolling motion. This study considered only one degree of freedom: rolling motion. To conduct this analysis, we developed a simplified time-domain simulator, using a single degree of freedom (SDoF) approach in the LMS Virtual Lab [27]. The simulator consisted of a linearized multi-body model (based on the CFD data), which included the forces generated by the flapping fins and the inertia of the mechanism and the entire vessel.

The fins were actuated according to the kinematics described in Section 3, and were phased to generate synergistic contributions, in order to damp the roll motion. We applied the forces from the CFD study to the center of mass and the center of pressure of the fins, and we found no significant differences in the outcome; therefore, in the end, we decided to apply the forces to the center of mass of the fins, because it remained fixed in time.

**Figure 36:**
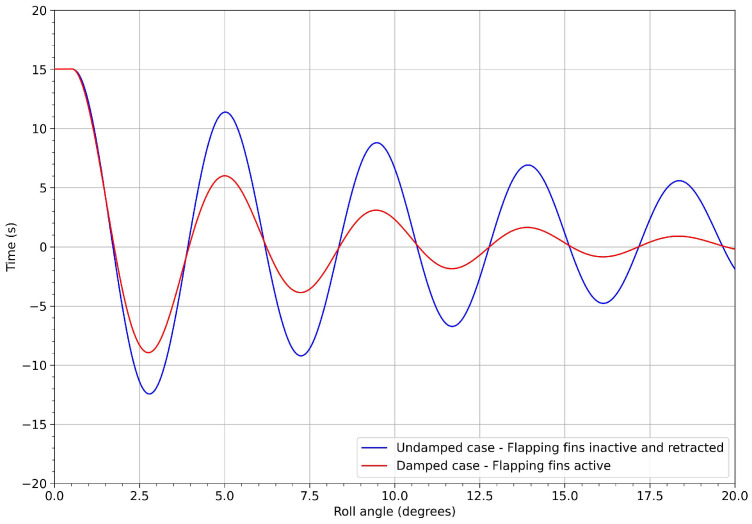
Roll angle evolution. The flapping frequency was equal to 0.2 Hz. Note that the starting roll angle was equal to 15 degrees (the maximum roll angle simulated using CFD).

**Figure 37:**
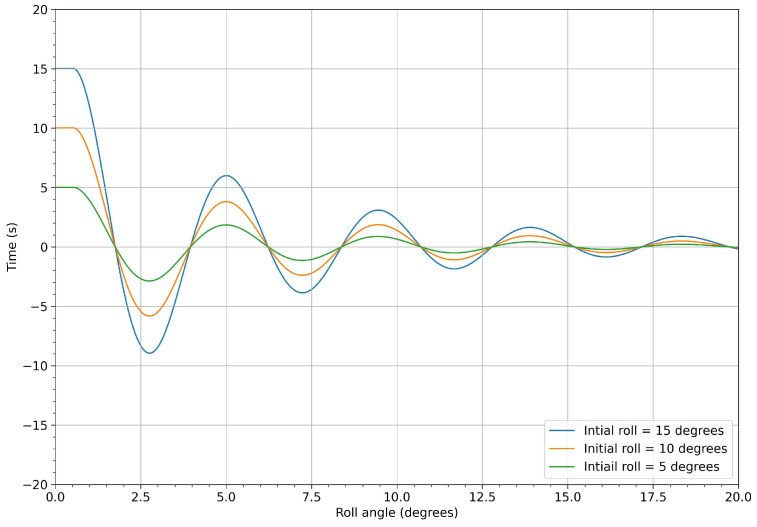
Roll angle evolution for three different initial conditions. The flapping frequency was equal to 0.2 Hz.

In Figure 36, we show the response of the undamped system (blue line), in terms of the rolling angle computed using CFD, and the contribution of inertia computed using the multi-body dynamics approach. This simulation was used as a reference to assess the effectiveness of the proposed mechanism in damping the rolling motion. In the same figure, the red line represents the response of the vessel with the stabilization system active. As can be seen in the figure, when the stabilization system was switch-on, the roll was effectively damped. After five seconds (one flapping cycle), the roll angle was reduced by approximately 40%, in reference to the undamped system. After 15 s (three flapping cycles), the roll oscillations were less than 2 degrees, which was deemed more than acceptable for passenger comfort. Figure 37 depicts the results for different roll initial conditions. These results confirm the effectiveness of the flapping system in damping the roll motion to acceptable levels.

It is worth noting that, for low roll angles (approximately below 2 degrees), the system was no longer effective, as the vessel started to roll in phase with the fins. This suggests the need to implement a control system to regulate the crank angular velocity or to limit the fins’ stroke.

## 8. Conclusions and Perspectives

The work presented in this manuscript is part of an ongoing multi-disciplinary effort to design an innovative mechanism for stabilizing recreational boats and yachts at anchor by using flapping fins. This multi-disciplinary study includes the design of the flapping mechanism, the hydrodynamic performance study, the structural analysis study, and the multi-body dynamics study.

It was found that the proposed mechanism and structural components withstand the static and dynamic loads generated by the flapping mechanism for frequencies up to 0.25 Hz. From the durability point of view, it was found that the fatigue life of the components was acceptable for frequencies up to 0.25 Hz.

The performance of the stabilization system was evaluated using a multi-body dynamics time-domain simulator that took, as input, the hydrodynamic forces and the inertia of the boat/mechanism obtained from CFD simulations and multi-body dynamics studies, respectively. The output of the simulator was the roll angle in function of time. From this study, it was found that the proposed mechanism effectively damped the roll motion. For a starting roll angle of 15 degrees, the roll motion was damped by as much as 40% in 5 s, and almost entirely damped after 15 s. Based on the results presented, the authors believe that it is feasible to build an operational proof-of-concept model of the flapping system, and to install it in a test boat.

Finally, the use of multi-body dynamics simulators was found to be a valuable tool for predicting the response of complex mechanical systems. The simulator used in this study was a simplified one, as we only considered a single degree of freedom (roll motion); however, it could be extended to more complex interactions. It is also envisaged to implement a control strategy to regulate the crank angular velocity or to limit the fins’ stroke.

## Figures and Tables

**Table 1:** Main characteristics and physical properties of the reference vessel. The vertical position of the center of gravity is measured in reference to the maximum depth of the vessel.

Parameter	Reference Value
Vessel displacement (*D*)	90,000 kg
Waterline length ($L_{WL}$)	22.4 m
Maximum waterline beam ($B_{WL}$)	5.6 m
Draft (*T*)	1.32 m
Vertical position of the center of gravity (KG)	2.46 m
Moment of inertia ($M_{xx}$)	600,000 kg·m${}^{2}$

**Table 2:** Main design specifications used in this study.

Vessel Characteristics	Value
Stabilization system available power ($P_{AVAIL}$)	5.0 kW per fin
Natural roll frequency of the vessel ($F_{ROLL}$)	0.2 Hz
Maximum surface area of a single fin	2.0 m${}^{2}$

**Table 3:** Main geometrical characteristics of the mechanism. The corrected angle $A_{f}$ was computed using the iterative process illustrated in Figure 7.

Geometrical Variable	Length (mm)	Angle ${(}^{\circ })$
Frame length (fixed component) $L_{1}$	488	-
Crank length $L_{2}$	210	-
Conrod length $L_{3}$	460	-
Rocker length $L_{4}$	264	-
Corrected angle $A_{f}$	-	$\approx 105.4^{\circ }$

**Table 4:** Physical properties of the phases used in this study. The surface tension was equal to 0.072 N·m${}^{-1}$.

Phase	Density ρ $\left(\frac{\mathrm{kg}}{m^{3}}\right)$	Kinematic Viscosity ν $\left(\frac{m^{2}}{s}\right)$
Water	998.3	$1.02\times 10^{-6}$
Air	1.2	$1.48\times 10^{-5}$

**Table 5:** Coefficients associated with Equation (6). The coefficients listed in this table corresponded to a frequency of 0.2 Hz.

Coefficient Index *n*	$a_{n}$	$b_{n}$
0	−1.383	–
1	−0.04723	−0.8749
2	0.1189	−0.03261
3	0.05942	0.01901
4	0.002404	0.01638
5	−0.00536	0.007733
6	−0.0029	−0.0002547
7	−0.0009761	−0.0014
8	0.0002754	−0.0005593

**Table 6:** Mean values of the hydrodynamic forces.

Frequency (Hz)	Mean $F_{x}$ (N)	Mean $F_{y}$ (N)	Mean $F_{z}$ (N)
1.0	6.5	17,500	1200
0.7	1.9	9200	880
0.5	2.6	5100	450
0.333	1.8	2100	100
0.25	0.8	1300	90
0.2	0.4	80	60

**Table 7:** Maximum values of the hydrodynamic forces.

Frequency (Hz)	Max. $F_{x}$ (N)	Max. $F_{y}$ (N)	Max. $F_{z}$ (N)
1.0	90	121,990	233,880
0.7	30	55,530	113,390
0.5	20	31,560	63,380
0.333	12	13,830	26,640
0.25	6	7900	15,800
0.2	3	5320	10,540

**Table 8:** Minimum values of the hydrodynamic forces.

Frequency (Hz)	Min. $F_{x}$ (N)	Min. $F_{y}$ (N)	Min. $F_{z}$ (N)
1.0	−42	−80,200	−12,7020
0.7	−39	−36,660	−64,700
0.5	−15	−20,720	−32,350
0.333	−2	−8990	−14,270
0.25	−1.5	−5180	−8090
0.2	−2	−3420	−5500

**Table 9:** Material physical properties. Standard steel.

Physical Properties	Reference Value
Young modulus (*E*)	$2\times 10^{11}$ Pa
Poisson module (ν)	0.346
Density (ρ)	7680 kg/m${}^{3}$
Yield strain ($\sigma_{s}$)	600–700 MPa
Ultimate strain ($\sigma_{u}$)	820 MPa

**Table 10:** Results of the structural analysis for each element of the mechanism at a frequency of 0.2 Hz.

Component	Maximum Stress (MPa)	Maximum Displacement (mm)	$\sigma_{s}$/$\sigma_{\max}$ Ratio
Crank	152	0.8324	3.95–4.61
Conrod	76.5	0.5674	7.84–9.15
Rocker	71.9	0.5179	8.34–9.74

## Data Availability

The data that support the findings of this study are openly available in figshare at https://doi.org/10.6084/m9.figshare.20662479, reference number [31].
